# A New Approach to the Fabrication of Memristive Neuromorphic Devices: Compositionally Graded Films

**DOI:** 10.3390/ma13173680

**Published:** 2020-08-20

**Authors:** Jong-Gul Yoon

**Affiliations:** Department of Physics and Electronic Materials Engineering, University of Suwon, Gyeonggi-do 18323, Korea; jgyoon@suwon.ac.kr

**Keywords:** compositionally graded oxide film, self-rectifying bipolar resistance switching, aerosol-assisted chemical deposition, short-term memory, ZnO-based memristor

## Abstract

Energy-efficient computing paradigms beyond conventional von-Neumann architecture, such as neuromorphic computing, require novel devices that enable information storage at nanoscale in an analogue way and in-memory computing. Memristive devices with long-/short-term synaptic plasticity are expected to provide a more capable neuromorphic system compared to traditional Si-based complementary metal-oxide-semiconductor circuits. Here, compositionally graded oxide films of Al-doped Mg*_x_*Zn_1−*x*_O (*g*-Al:MgZnO) are studied to fabricate a memristive device, in which the composition of the film changes continuously through the film thickness. Compositional grading in the films should give rise to asymmetry of Schottky barrier heights at the film-electrode interfaces. The *g*-Al:MgZnO films are grown by using aerosol-assisted chemical vapor deposition. The current-voltage (*I-V*) and capacitance-voltage (*C-V*) characteristics of the films show self-rectifying memristive behaviors which are dependent on maximum applied voltage and repeated application of electrical pulses. Endurance and retention performance tests of the device show stable bipolar resistance switching (BRS) with a short-term memory effect. The short-term memory effects are ascribed to the thermally activated release of the trapped electrons near/at the *g*-Al:MgZnO film-electrode interface of the device. The volatile resistive switching can be used as a potential selector device in a crossbar memory array and a short-term synapse in neuromorphic computing.

## 1. Introduction

One of the critical issues of current digital computing using conventional von-Neumann architecture is processing bottlenecks that are caused by extensive data transfer between the central processing unit and the memory unit for data-intensive tasks [1]. Neuromorphic computing architectures are the alternatives to the existing computing system [2], which are inspired by parallel information processing in the human brain with high density neural networks and ultra-low power consumption [3]. To mimic the human brain, neuromorphic circuits that can process information with massive parallelism and ultra-low power dissipation should be realized. Thus, the main technological challenges of neuromorphic computing are the development of memory devices serving the role of synaptic links and/or neuron elements and computing architectures that promise advanced computing functionality with high scalability and low-power operation [4,5,6,7,8].

Significant effort has been devoted to realizing the functionalities of neurons and synapses for neuromorphic computing using emerging nonvolatile memories (NVMs) [9]. Recently, several memristive NVMs, including resistive random-access memory and phase-change memory, have emerged [9,10,11,12,13]. Memristive devices, which exhibit history-dependent conductivity modulation [4,8,9,10,11,12,13], are more efficient than traditional Si-based complementary metal-oxide semiconductor (CMOS) circuits [6] in providing more capable neuromorphic systems. They can store information at the nanoscale in an analogue way and allow in-memory computing, which is a major approach toward energy-efficient computing paradigms beyond the von-Neumann architecture [14,15]. The resistance/conductance states of the memristive devices are used for storing the synaptic weights and performing the associated in-memory computational tasks in artificial neural networks. Along with the memristive NVMs, volatile memristors [16,17,18] also have attracted strong interest as artificial synapses to mimic short-term memory phenomena in the brain. Short-term synaptic plasticity (STP) refers to a type of neural plasticity where the effective synaptic strength (connection strength) between two neurons changes dynamically in a short time as a result of recent activity of the neurons. These temporary changes are restored in the absence of input. The short-term memory effects can provide a method of implementing the biological synaptic behavior, such as synaptic influx and extrusion of the Ca^2+^ ion [19]. It has been demonstrated that a diffusive memristor and its dynamics can emulate both the short- and long-term plasticity of biological synapses [8]. However, significant improvements in the characteristics of the memristive devices are needed to incorporate such synaptic devices into large-scale neuromorphic systems. Since the implementation of synaptic efficacy and the plasticity of the memristive device that refer to the generation of a synaptic output based on the incoming neuronal activation and the ability of the synapse to change its weight, respectively, are entirely dependent on the conductance changes upon applying an appropriate write/read voltage signal, a precise modulation of the resistance/conductance over a wide dynamic range is a very important and quite challenging task [20].

Here, a new approach to the control of electrical transport properties of oxide thin films for memristive devices is reported by fabricating compositionally graded Al-doped Mg*_x_*Zn_1−*x*_O (*g*-Al:MgZnO) films. The deposition of *g*-Al:MgZnO films using aerosol-assisted chemical deposition (AACVD) are described in detail, including the method of controlling the concentration of elements in the films continuously for compositional grading. The composition and structure of the films are analyzed by secondary ion mass spectroscopy (SIMS), X-ray photoelectron spectroscopy (XPS), and X-ray diffraction (XRD) θ–2θ scanning. The *g*-Al:MgZnO films show asymmetric hysteretic current-voltage (*I*-*V*) and capacitance-voltage (*C*-*V*) characteristics, resulting in self-rectifying bipolar resistance switching (BRS). The self-rectifying BRS with variable conductance can be used for memristive neuromorphic devices as artificial synapses, as well as a select device in the cross-bar structure of memristors. Endurance and retention performance tests for the devices of metal-semiconductor (*g*-Al:MgZnO)-metal (MSM) structure show quite reliable short-term memory effects. Electrically driven electron trapping at the interface and detrapping effects are discussed in conjunction with the short-term memory effects.

## 2. Materials and Methods

### 2.1. Compositionally Graded g-Al:MgZnO Films

Band gap grading via compositional grading is a powerful tool for engineering the electrical transport properties of semiconductors [21]. Al*_x_*Ga_1−*x*_As is a good example of such graded band gap systems [22,23] in which the composition of the system gradually changes from that of a small-band-gap material, such as GaAs (1.441 eV), at one end to that of a large-band-gap material, such as AlAs (2.12 eV), at the other. These structures have stable characteristics and are widely used in photovoltaics [24]. Electrons and holes in the system experience different electric forces due to the quasi-electric field induced by the graded band gap, so the transport properties of the two types of carriers can be tuned independently [22,23].

ZnO is a well-known oxide semiconductor with a wide band gap of about 3.2 eV [25]. The band gap of ZnO can be controlled to have a smaller or larger band gap by replacing the Zn element with other elements such as Cd or Mg [26,27]. Most previous studies on ZnO-based systems are related to the optoelectronic properties of the materials due to the large exciton binding energy of ZnO [24]. Here, compositionally graded film, *g*-Al:MgZnO, is deposited to fabricate a memristive neuromorphic device by modifying the electrical transport properties. MgO is alloyed with increasing concentration during the growth of the film to obtain a graded band gap across the film thickness. Since Mg alloying with ZnO can cause a conductivity decrease by the formation of acceptor-like compensating intrinsic defects, such as zinc vacancies [26], Al-dopant is continuously increased toward the surface of the film to tune the conductivity of the graded film.

### 2.2. AACVD of g-Al:MgZnO Films

**Figure 1:**
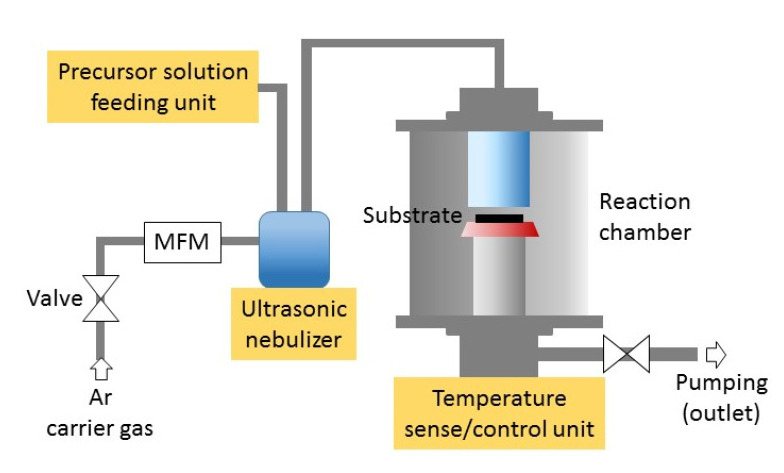
Schematics of an AACVD system. MFM: Mass flow meter.

Advances in thin film technology have enabled the fabrication of well-controlled graded band gap semiconductor systems [22,23]. Regarding oxide materials, deposition of epitaxial films with graded composition is possible when the constituent materials of the films are isomorphic [28,29,30]. During this experiment, *g-*Al:MgZnO films are deposited using AACVD, as shown schematically in Figure 1. AACVD has advantages over a conventional CVD (Chemical Vapor Deposition) technique, especially in the case of no suitable precursor materials for the gaseous state being available [31,32]. To provide an example, if the vapor pressure of metal-organic materials for CVD is too low, conventional CVD is difficult to apply for growing thin films because the gaseous state of the materials is not available. However, transferring the precursor materials to the substrate in the form of an aerosol is possible even if the vapor pressure of the material is low, so the selection range of the source materials is wide, reducing the overall cost for deposition [31,32].

The composition control of *g*-Al:MgZnO films can be achieved by changing the relative concentrations of the source materials in the precursor solution during AACVD. The precursor solution for MgO is supplied continuously to the flask containing a Zn-precursor solution of initial volume *V*_0_*^Zn^* at a constant rate of *V*_0_*^Mg^/t_Mg_* during the deposition using a syringe pump (precursor solution feeding unit), where *t_Mg_* is the total supply time of the Mg-source solution of volume *V*_0_*^Mg^*. Molar concentration of each precursor solution is the same. During the deposition, the concentration of Mg, *c_Mg_*, in the mixed precursor solution continues to increase over time due to the transfer of generated aerosol and can be estimated by the following equation:

(1) $$c_{Mg}\left(t\right)=\frac{V\left(t\right)-V^{Zn}\left(t\right)}{V\left(t\right)}$$

where *V*(*t*) and *V^Zn^*(*t*) are the volumes of the mixed precursor solution and that of the Zn-source solution, respectively, given as a function of time by:

(2) $$V\left(t\right)=V^{Zn}\left(t\right)+V^{Mg}\left(t\right)=\left(\frac{V_{0}^{Mg}}{t_{Mg}}-K\right)t+V_{0}^{Zn}$$

(3) $$V^{Zn}\left(t\right)={\left(V_{0}^{Zn}\right)}^{\left(\frac{Kt_{Mg}}{V_{0}^{Mg}-Kt_{Mg}}+1\right)}{\left[\left(\frac{V_{0}^{Mg}}{t_{Mg}}-K\right)t+V_{0}^{Zn}\right]}^{-\left(\frac{Kt_{Mg}}{V_{0}^{Mg}-Kt_{Mg}}\right)}$$

where *K* is the consumption rate of the precursor solution due to the transfer of aerosol to the reaction chamber (see Appendix A).

Compositionally graded *g*-Al:MgZnO films were deposited on platinized Si substrate (Pt/Ti/SiO_2_/Si) at 420 °C. A ZnO buffer layer of about 200 nm thickness was deposited for the growth of *g*-Al:MgZnO film [33]. The *g*-Al:MgZnO film of about 250 nm thickness was deposited subsequently on top of the ZnO layer under the same deposition condition. The precursor solution for the deposition of the *g*-Al:MgZnO thin film was prepared by dissolving Zn-acetate (Zn(CH_3_COO)_2_) and Mg-acetate (Mg(CH_3_COO)_2_·4H_2_O) in 2-methoxyethanol, respectively, with the same concentration of 0.01 M. Al-acetylacetonate (Al(C_5_H_7_O_2_*)*_3_) was used as a source material for the Al dopant. Aerosol of the precursor solution was generated by an ultrasonic nebulizer operating at a frequency of 1.6 MHz and transferred to the reaction chamber by an Ar carrier gas flowing at a rate of 400 mL/min. The pressure in the reaction chamber was maintained at atmospheric pressure during deposition. The transferred aerosol was pyrolyzed on a heated substrate to form a solid thin film. After deposition, post-annealing was carried out at 500 °C for 30 min.

### 2.3. Analysis of g-Al:MgZnO Films and Electrical Measurements

Compositional grading in the films was investigated by analyzing the depth profiles of the film composition using time-of-flight (TOF) SIMS (Münster, Germany) as a function of sputtering time. During the TOF-SIMS measurement, Bi_1_^+^ ion beams of 25 KeV were used as a primary ion and positively charged secondary ions were analyzed. The analysis area was set at 80 × 80 µm^2^. The sputter ions were O_2_^+^ with their energy set at 2 keV. XPS depth profiling also was carried out to confirm the graded composition of the *g*-Al:MgZnO/ZnO film. The crystalline property and graded lattice structure of *g*-Al:MgZnO film were examined by XRD θ–2θ scan.

The electrical properties of *g*-Al:MgZnO/ZnO heterostructure film were investigated by measuring *I*-*V* characteristics with varying the maximum applied voltage, *V*_max_. Concerning the measurements, an Au top electrode of 100 µm diameter was deposited on the surface of the film using a vacuum evaporator. The driving voltage for the *I*-*V* measurements was applied to the Au top electrode of the *g*-Al:ZnMgO layer with the bottom Pt electrode grounded. The typical voltage sweep rate was |*dV/dt*| *=* 0.2 V/s. Changes in the conductance of the device, which corresponded to the changes in synaptic weight or synaptic strength in the neural system, were observed by measuring the *I*-*V* characteristics with a different *V*_max_ or repetition rates of applied voltage. *C*-*V* curves for the devices were measured at different frequencies to investigate the effects of charge trapping near/at the interfaces of the film. Performance reliability as a memristive device was tested by measuring endurance and retention characteristics.

## 3. Results and Discussion

### 3.1. Analysis of Composition and Structure of g-Al:MgZnO Films

**Figure 2:**
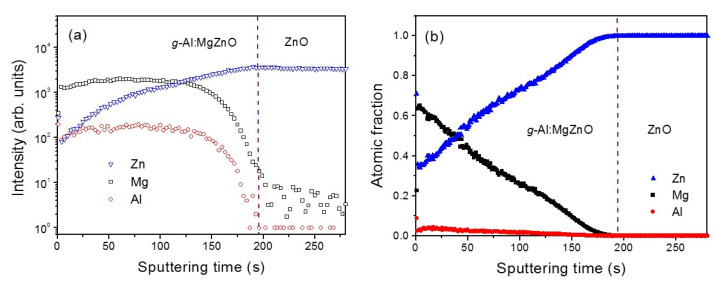
(**a**) SIMS depth profiles of the elements and (**b**) the atomic fractions estimated from the SIMS results for the *g*-Al:MgZnO/ZnO heterostructure film.

The graded composition of a *g*-Al:MgZnO film is confirmed by depth profile analysis using TOF-SIMS. The yield intensity of SIMS as a function of sputtering time is shown in Figure 2a. The sample was profiled from the surface of the film. A gradual increase in the intensity of the Zn element is distinctive toward the bottom interface of the film. An approximate quantification of the atomic fraction of each element is carried out using relative sensitivity factors derived from a reference Al_0.03_Mg_0.58_Zn_0.39_O polycrystalline film whose composition was determined by X-ray fluorescence analysis. Shown in Figure 2b, the atomic fractions of Mg and Al are continuously increasing while that of Zn is decreasing toward the film’s surface. Seen at the uppermost surface of the *g*-Al:MgZnO layer, the composition is analyzed to be around Al_0.03_Mg_0.64_Zn_0.33_O. XPS analysis (not shown) confirmed nearly the same quantitative compositional gradient and provided additional information on oxygen distribution that decreased toward the bottom ZnO layer. Thus, band gap grading from a small to large band gap is expected toward the surface of the *g*-Al:MgZnO layer because Mg alloying is known to increase the band gap of ZnO [26,27]. The depth profile analysis using SIMS and XPS confirms well-controlled deposition of the compositionally graded layer by AACVD.

**Figure 3:**
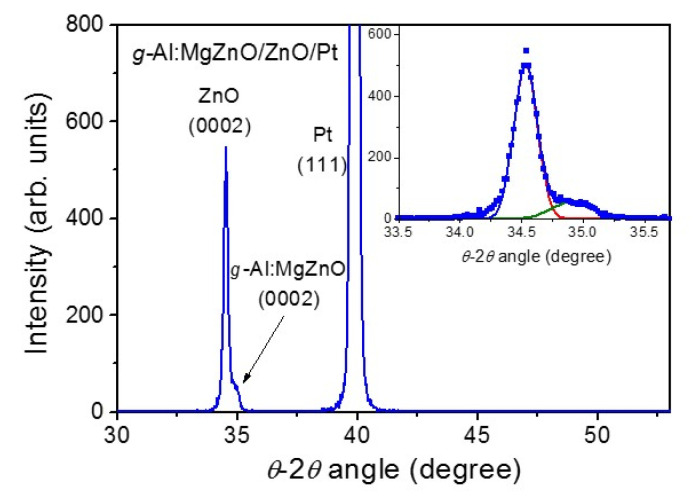
The XRD result for *g*-Al:MgZnO/ZnO heterostructure film. The inset is a Gaussian fitting of the XRD peaks for ZnO (0002) and *g*-Al:MgZnO (0002).

A compositionally graded *g*-Al:MgZnO film is expected to have different lattice constants across the film thickness due to the compositional grading. Figure 3 shows the XRD θ–2θ scan for the *g*-Al:MgZnO/ZnO heterostructure film. The XRD pattern only shows a distinct (0002) peak of hexagonal wurtzite structure of ZnO at 2θ value of 34.5° in the measured range. It should be noted that a broad shoulder-like feature on the right side of the ZnO (0002) XRD peak is observable (see the inset for the XRD peak-fitting). This result indicates a graded lattice structure of *g*-Al:MgZnO film where the lattice constant along the film thickness is decreasing continuously, in consistent with the expectation of the decreased *c*-axis lattice constant of ZnO upon alloying with MgO [25,26,27,34,35]. It is interesting that there is no evidence of the formation of a cubic MgZnO phase, even with the composition of Mg in the graded layer being increased up to *x* = 0.65. The single crystalline ZnMgO films with *x* > 0.32 have been reported to show a cubic phase following the structure of MgO [34,35]. It is inferred that the in-plane strain induced by the underlying layer of the hexagonal wurtzite structure may suppress the formation of a cubic MgZnO phase in the graded layer. Additionally, a Bragg condition for diffraction would be weakly satisfied in the short ranges due to a continuous change in the lattice parameters, which hinders the appearance of distinct XRD peaks of a cubic phase in the graded layer.

### 3.2. I-V and C-V Characteristics of g-Al:MgZnO Films

**Figure 4:**
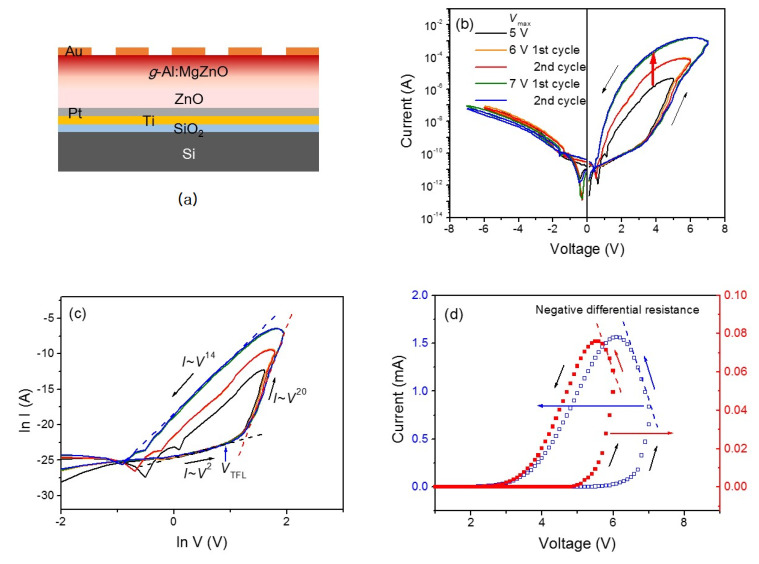
(**a**) Metal-semiconductor-metal (MIM) structure of device based on *g*-Al:MgZnO/ZnO heterostructure film; (**b**) *I*-*V* characteristics of the device with different *V*_max_ with a voltage sweep from 0 V to +*V*_max_, +*V*_max_ to −*V*_max_ followed by −*V*_max_ to 0 V; (**c**) the logarithmic plots of the *I*-*V* curves showing a power-law dependence, *I*~*V^α^*; (**d**) negative differential resistance around *V*_max_ upon changing the voltage sweep direction to *dV/dt* < 0.

The schematic of the *g*-Al:ZnMgO/ZnO heterostructure device is shown in Figure 4a. The functional core based on a *g*-Al:ZnMgO/ZnO heterostructure film is effectively considered as a semiconductor layer with a graded wide band gap. It should be noted that the top electrode is Au whose work function (~5.1 eV) is lower than that of the Pt bottom electrode (~5.6 eV). Thus, the device has asymmetric Schottky contacts with different Schottky barriers at the film-electrode interfaces. Figure 4b is the *I*-*V* characteristics of the device with a different *V*_max_. The measurements were carried out by sweeping the DC bias from 0 V to +*V*_max_, +*V*_max_ to −*V*_max_ followed by −*V*_max_ to 0 V. The curves show interesting features to note: They reveal a significant hysteretic behavior, which is a signature of an memristor, and a rectifying characteristic. The counterclockwise hysteretic behavior is observed only in the positive bias region and is quite reproducible following the same *I*-*V* curve without much deviation. Additionally, *I*-*V* curves show a non-zero crossing behavior, indicating the existence of an internal field or the presence of some parasitic capacitance. The low-resistance state (LRS) current and the width of hysteresis (hysteresis window) increases with the increase in *V*_max_. This type of response corresponds to synaptic weight modulation by input signals in a neural network. Concerning the crossbar array of memristors, the input signal and synaptic weight are regarded as the applied input voltage and conductance of the corresponding memristor, respectively.

The rectifying characteristic of the device is attributed to the graded band gap of *g*-Al:ZnMgO, causing highly asymmetric Schottky barrier heights at the electrode contacts [29]. The current in the positive bias region increases rapidly above a voltage of ~3.5 V and *I*-*V* curves become quite asymmetric, resulting in the rectifying behavior. Interestingly, the voltage dependence of the hysteretic current in the positive bias region can be fitted to a power law, *I*~*V^α^*, as shown in Figure 4c, where the exponent α varies in the range of 2–20 depending on the bias voltage region and voltage sweep direction. The power-law behavior of *I-V* characteristics is usually modeled in terms of a space-charge-limited conduction (SCLC) mechanism controlled by the trapped charge density [36]. Upon increasing the bias voltage, the accept-like trap states [28] in the graded layer are occupied by injected electrons and trap*-*filled limited (TFL) conduction (α > 2) showing a rapid increase in current would induce LRS at the crossover voltage to TFL conduction, *V*_TFL_, which can be considered as the set voltage of switching to LRS. The resultant self-rectifying characteristic of this memristive device can suppress the so-called sneak-path problem in the crossbar synaptic array [37].

A negative differential resistance (NDR) also is observed when the voltage sweep direction is changed (*dV/dt* < 0) after reaching *V*_max_ in the positive bias region, as shown in Figure 4d. It shows a continuing increase of current, even when the bias voltage is decreasing. The larger the *V*_max_, the more prominent the NDR. The appearance of NDR is attributed to a possible discharge process in the thin film. Under high bias voltages, trapped electrons are detrapped by the field-assisted Poole-Frenkel (PF) effect [38] and the transient increase in carrier density can result in the abnormal current increase upon changing the voltage sweep direction from positive to negative. Since the detrapping rate of electrons may increase with *V*_max_, NDR would become more prominent for a larger *V*_max_.

**Figure 5:**
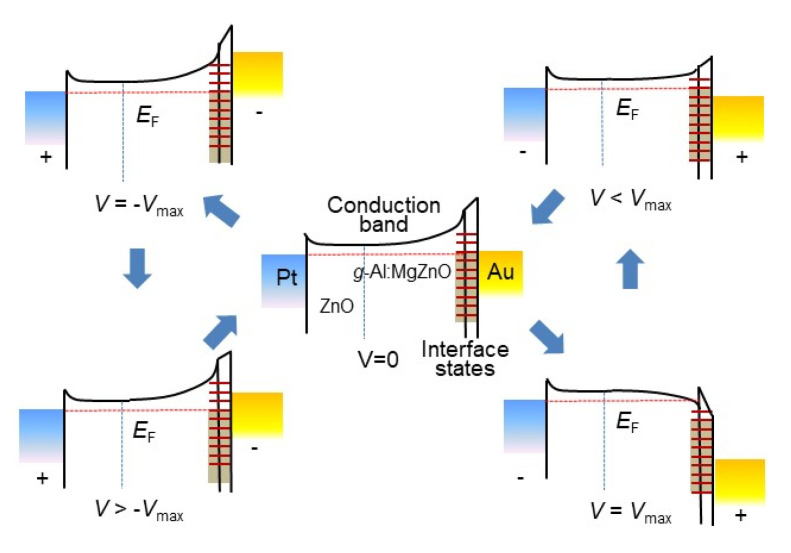
Schematics of charge trapping/detrapping at interface states during the bias voltage sweep (0 V→ *V*_max_ → −*V*_max_ → 0 V) and modified Schottky barriers by Fermi level pinning. Interface localized states are indicated in short red lines at the interface and those in the shadowed region are filled states.

The highly voltage-dependent hysteretic *I-V* characteristics of the device, as revealed in Figure 4b–d, can be explained by charge trapping/detrapping and Fermi level pinning, which occurs upon interface formation and/or incorporation of chemical impurities [39]. Occurring at the top interface between *g-*Al:ZnMgO and the Au top electrode of the device, the Schottky barrier height is determined by the occupation of interface localized states, thus depends on charge trapping/detrapping [40,41]. When in equilibrium, a higher band gap of the uppermost *g-*Al:ZnMgO layer should result in a higher Schottky barrier at the interface between the graded layer and the Au top electrode, as shown in Figure 5. During the positive bias region with *dV/dt* > 0, thermally generated or injected electrons begin to occupy accept*-*like interface states [28] and the Fermi level is pinned at the highest level of the interface states at *V*_max_, resulting in a lower Schottky barrier height. Additionally, trapped charges near/at the interface may cause narrowing of the Schottky barrier width, facilitating LRS. Thus, LRS is acquired and maintained in the positive bias region during *dV/dt* < 0. On the other hand, modulation of the Schottky barrier height and width at the interface between the ZnO layer and the Pt bottom electrode may not be significant due to the smaller band gap of ZnO compared to that of the uppermost layer of *g-*Al:ZnMgO. Additionally, the density of the charge trapping centers at the bottom interface is expected to be much less than that at the top interface. It is believed that the self-rectifying memristive behavior is largely determined by the interface between the *g-*Al:ZnMgO layer and the Au top electrode.

**Figure 6:**
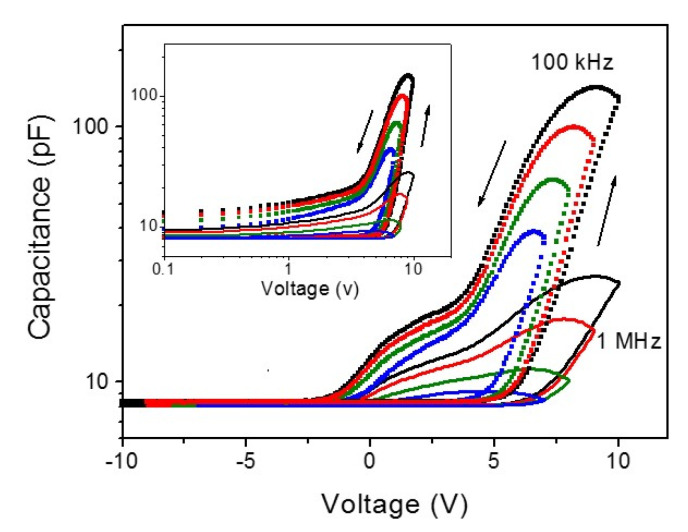
*C-V* characteristics of *g*-Al:MgZnO/ZnO heterostructure film for two different measuring frequencies of 100 kHz and 1 MHz with a different *V*_max_. The inset is double logarithmic plots of the *C-V* curves, showing a power*-*law behavior.

However, referring to the *I-V* curves in Figure 2b, it seems that LRS returns to a high*-*resistance state (HRS) in a short time if the applied voltage is removed. This means that Fermi level pinning is not so strong and the Schottky barrier can be tuned by the occupation level of the interface states. When the bias voltage is removed or becomes negative, electrons will be gradually detrapped from the interface states and Fermi level pinning will occur at lower energy levels at the interface, resulting in HRS due to the change in the Schottky barrier height and width. The occupation of interface-localized states and the accumulation of charge near/at the interfaces may induce an interfacial polarization and cause an increase in the dielectric constant of the layer in the positive bias region by Maxwell-Wagner effects [42]. Actually, *C-V* measurements show significant increases in the capacitance and the hysteresis that depends on *V_max_* in the positive bias region, as shown in Figure 6. Considering the inset, the counterclockwise hysteretic *C-V* curves are plotted in double logarithmic scale to compare with the *I-V* curves shown in Figure 4c. The curves also follow power-law dependences in different regions during the voltage sweep, indicating that the interfacial polarization is closely related to the current through the heterostructure film. The hysteretic behavior continues down to the negative bias region, suggesting that the charge trapping mechanism throughout the *g-*Al:ZnMgO layer and interfaces is responsible for the *C-V* hysteresis. The *C-V* hysteresis window, Δ*V,* can be used to estimate the density of the charge trapping using the equation, *N_trapped_* = *C_film_*Δ*V/q,* where *C_film_* is the capacitance of the *g*-Al:ZnMgO/ZnO heterostructure film and *q* is the elementary charge. Figure 6 shows that the Δ*V* is increasing as the *V*_max_ increases at a given capacitance value of the film, indicating that the density of the trapped charge as well as the occupation level of the interface states are quite dependent on *V*_max_ and relevant to the hysteretic behaviors of the *I-V* and *C-V* characteristics.

### 3.3. Effects of Bipolar Voltage Pulse Trianing

**Figure 7:**
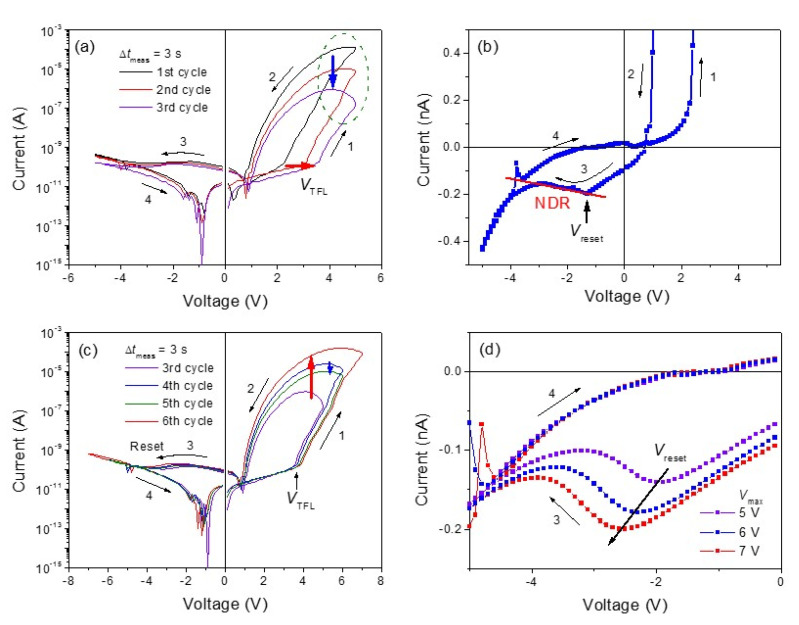
(**a**) Relaxation behavior of the BRS characteristics of the *g*-Al:MgZnO/ZnO heterostructure device just after 500 cycles of bipolar voltage pulse training. (**b**) A linear scale plot of the *I-V* curve for the 1st cycle of the *I-V* measurements showing an NDR above the reset voltage, *V*_reset_. (**c**) *V*_max_ dependence of *I-V* characteristics of the film after the relaxation. Δ*t*_meas_ is the time interval between the measurements. (**d**) Shift of *V*_reset_ to larger negative voltages for higher *V*_max_.

It is required that the conductivity of device can be modulated by the application of successive stimulating signals to emulate synaptic efficacy and plasticity of neurons. *I-V* characteristics of a *g-*Al:MgZnO/ZnO heterostructure device are measured after bipolar voltage pulse training to investigate the effects of training on the conductivity modulation. Regarding nonvolatile memories, the training process would be equivalent to applying potentiating and depressing input-signals repeatedly to the device. Bipolar square wave voltage pulses of ±7 V are applied to the device at a frequency of 100 Hz. Figure 7a,c shows the modified *I-V* characteristics measured just after 500 cycles of the electrical pulse training and the subsequent measurements followed by applying different *V*_max_, respectively. Compared to the *I-V* curves measured before the electrical training (refer to Figure 4b), *I-V* curves after the electrical training show much clearer bipolar resistive switching (BRS) behavior with a non-zero crossing characteristic. The *I-V* curve plotted in linear scale (Figure 7b) shows a clear signature of NDR in the negative bias region where the reset process to HRS is driven by charge detrapping from the interface states [41]. The overall *I-V* characteristics show BRS with eightwise polarity, which is ascribed to the change in the Schottky barrier height and width by the trapping/detrapping effects at the interface defect states. The self-rectifying characteristic is enhanced after the electrical training such that the rectifying ratios increase up to 10^5^ in the measured voltage range. It should be noted that the self-rectifying BRS behavior is obtained without any electroforming process.

The most intriguing effects of the electrical training are the modification of *I-V* characteristics and the appearance of a relaxing behavior of the *I-V* curve in the positive bias region. The *I-V* curve, just after the electrical training (1st cycle in Figure 7a), shows a narrow hysteresis window with high current levels at LRS for the same *V*_max_. Multiple applications of bipolar voltage pulses seem to lower the *V*_TFL_ and, subsequently, increase the overall current levels. Thus, the onset voltage to LRS is lowered and the conductivity of the device is increased. This result implies that a successive application of constant voltage pulses can facilitate LRS or the high conductance state of the device, which should correspond to stimulation-induced synaptic potentiation. However, such effects of the electrical training begin to relax in a short time: *I-V* characteristics of the device gradually change in such a pattern of increasing *V*_TFL_ and a decrease in the current levels of LRS as the measurement is repeated, as indicated in Figure 7a (2nd and 3rd cycles) with arrows. Interestingly, NDR is not observed in the positive bias region just after the electrical training but appears again as the relaxation proceeded, as indicated with the dashed circle in Figure 7a. It seems that, just after the electrical training, most of the traps, including the interface states, are filled with electrons. However, the filled traps can be detrapped by a voltage sweep during the subsequent *I-V* measurements and the reappearance of NDR. Changes in the trapped charge density may be responsible for the relaxed behavior of the *I-V* characteristics.

The *V*_max_ dependence of the *I-V* characteristics after the relaxation of the *V*_TFL_ are shown in Figure 7c. The *I-V* curves show clear self-rectifying BRS behaviors with enhanced hysteresis windows compared to those before the electrical training. The relaxing behavior of the current levels in LRS is still observable for the same *V*_max_, as indicated with the downward arrow in Figure 7c (curves for 4th and 5th cycles). On the other hand, there is little change in the *I-V* characteristics in the negative bias region. Interestingly, the reset voltage *V*_reset_ tends to shift to higher negative voltages as *V*_max_ increases, as shown in Figure 7d. The shift of the *V*_reset_ has been reported and ascribed to charge detrapping [41], since increasing *V*_max_, interface states will be occupied by more electrons. The electrons can be detrapped when the voltage sweeps from zero to −*V*_max_, resulting in larger currents and a higher transition voltage *V*_reset_ for NDR. The reset process corresponds to synaptic depression if long-term potentiation has been achieved in the device. However, the *g*-Al:MgZnO/ZnO heterostructure device allows only short-term plasticity due to the relaxation effect, as will be discussed later in Section 3.4.

It is inferred that distribution of charge trapping defects may not be homogeneous due to the graded composition of the *g*-Al:MgZnO layer. It has been reported that the Mg alloying produces deep defect levels and causes a strong carrier compensation effect by trapping charges [43,44]. Defects such as Zn vacancies [43], as well as oxygen vacancies [44], have been reported to be responsible for the charge trapping centers. Such inhomogeneous distribution of charge trapping centers may be responsible for the asymmetric relaxation effects after the bipolar voltage pulse training. This relaxing behavior should be related to the short-term synaptic plasticity of the device.

### 3.4. Reliability Tests for Memristor

**Figure 8:**
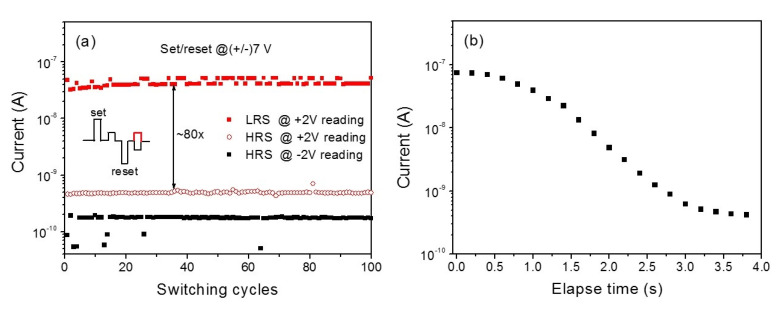
(**a**) Endurance performance of the memristive device based on *g*-Al:MgZnO/ZnO heterostructure film. (**b**) Retention characteristic of the device showing a short-term memory effect.

To evaluate the memory performance of the device, the endurance property, the LRS:HRS current ratio, and the data retention ability should be tested. The endurance and retention performance of the device was investigated after the electrical training which induced clear BRS characteristics, as observed. The endurance performance of the device is shown in Figure 8a. The test was conducted by applying a sequence of voltage pulses for set/reset and reading the resistance states of the device. The schematic voltage pulse train is shown in the inset of Figure 8a. The set/reset pulse voltage was +/− 7 V and the reading pulse voltage was 2 V with the pulse width of 10 ms. The reading pulse voltage was chosen at the voltage below *V*_TFL_ (refer to Figure 7a,c) so as not to affect the trapped charge profile. The LRS: HRS current ratio is estimated to be about 100 at the measurement condition of the positive pulse reading. The relative variation of the currents, both at HRS and LRS, is very low and quite stable. The retention property also was tested via measuring currents by applying periodic reading voltage pulses of 2 V with an interval of 200 ms. Figure 8b shows the retention characteristic of the device. After the set process, LRS decays to HRS in 3 s, showing a short-term memory effect, while the HRS current does not change. This result indicates that interface-type memristive devices may show only short-term plasticity.

The short-term memory effect should be related to charge detrapping from the interface states and the subsequent change in the Schottky barrier height and width. The charge detrapping should be thermally activated and the Fermi level pinning occur at lower energy levels at the interface, which would result in an HRS with an increased Schottky barrier height and width. It has been reported that interface-type resistive switching devices show a volatile or short-term memory effect [45]. However, different mechanisms for different systems have been proposed to explain the short-term memory effects [8,16,17,18]. Although the hardware implementations of artificial neural networks have focused mainly on long-term plasticity, short-term plasticity also is worthy of consideration for enhancing discrimination of spatiotemporal stimuli [46]. The volatile resistive switching also can be used as a potential selector device in a crossbar memory array and a short-term synapse in neuromorphic computing [18].

## 4. Summary and Conclusions

A precise modulation of the resistance/conductance of memristive devices with an electrical signal is one of the important requirements for the implementation of synaptic efficacy and plasticity of the device for neuromorphic computing. Considering this, a new approach to the control of electrical transport properties of materials for memristive devices is proposed by investigating the electrical properties of compositionally graded Al-doped Mg*_x_*Zn_1−*x*_O (*g*-Al:MgZnO) films. Details of the composition control of the films are described and the graded composition of the film is confirmed by depth profile analysis of the composition and XRD θ–2θ scan. The graded composition of the films is found to induce asymmetric electrical transport properties as well as structural asymmetry. Interestingly, *I-V* characteristics show highly rectifying and counterclockwise hysteresis in the positive bias region. Such electrical properties can be considered as a signature of a self-rectifying memristor. The power-law behavior of the *I-V* characteristics, *I*~*V*^α^, and NDR in the positive bias region is discussed in terms of an SCLC mechanism controlled by trapped charge density and field-assisted PF emission at high bias voltages. The counterclockwise hysteresis behavior also is significantly dependent on *V*_max_, which is required for the implementation of the synaptic efficacy and plasticity of memristive devices. The highly voltage-dependent hysteretic *I-V* characteristics of the device are explained in terms of charge trapping/detrapping and Fermi level pinning at the interface localized states, as well as by asymmetric distribution of structural defect actions. Furthermore, bipolar voltage pulse training significantly improves the self-rectifying BRS characteristic of the device, which would be related to the modulation of synaptic weight in the learning process of the neuromorphic system. Endurance performance of the device is shown to be quite stable with a high LRS: HRS current ratio of about 100. On the other hand, the currents in LRS, just after the electrical training, are found to decay to HRS in a short time. The short-term memory effect should be related to the charge trapping/detrapping mechanism at the top interface and is believed to provide the short-term synaptic plasticity of the memristive device based on compositionally graded films. The volatile resistive switching also can be used as a potential selector device in the crossbar memory array and short-term synapse in neuromorphic computing. The interface-type self-rectifying BRS devices exhibiting multilevel resistance switching can be a key component for neuromorphic device applications.

## Appendix A

The calculation of the concentration of Mg in the mixed precursor solution, is given as follows: When the precursor solution of volume *V*_0_*^Mg^* for MgO is supplied continuously at a constant rate of *V*_0_*^Mg^/t_Mg_*, where *t_Mg_* is the total supply time of Mg-source solution, to the flask containing Zn-precursor solution of initial volume *V*_0_*^Zn^*, the concentration of Mg-source, *c_Mg_*, in the mixed precursor solution during the film deposition is given as a function of deposition time *t* as follow:

(A1) $$c_{Mg}\left(t\right)=\frac{V\left(t\right)-V^{Zn}\left(t\right)}{V\left(t\right)}$$

where *V*(*t*) and *V^Zn^*(*t*) are the volumes of the mixed precursor solution and that of Zn-source solution, respectively. *V*(*t*) and *V^Zn^*(*t*) are given as a function of time by

(A2) $$V^{Zn}\left(t\right)=V_{0}^{Zn}-\int_{0}^{t}s\left(t^{\prime }\right)V^{Zn}\left(t^{\prime }\right)dt^{\prime }$$

(A3) $$V^{Mg}\left(t\right)=\frac{V_{0}^{Mg}}{t_{Mg}}t-\int_{0}^{t}s\left(t^{\prime }\right)V^{Mg}\left(t^{\prime }\right)dt^{\prime }$$

where, *s**(t*) is the consumption rate of the mixed precursor solution due to the generation of aerosol and given by

(A4) $$s\left(t\right)=\frac{K}{V^{Zn}\left(t\right)+V^{Mg}\left(t\right)}$$

*K* is a constant representing the consumption rate of the precursor solution due to the transfer of aerosol to the reaction chamber. Solving the equations, we obtain the solution

(A5) $$V\left(t\right)=V^{Zn}\left(t\right)+V^{Mg}\left(t\right)=\left(\frac{V_{0}^{Mg}}{t_{Mg}}-K\right)t+V_{0}^{Zn}$$

(A6) $$V^{Zn}\left(t\right)={\left(V_{0}^{Zn}\right)}^{\left(\frac{Kt_{Mg}}{V_{0}^{Mg}-Kt_{Mg}}+1\right)}{\left[\left(\frac{V_{0}^{Mg}}{t_{Mg}}-K\right)t+V_{0}^{Zn}\right]}^{-\left(\frac{Kt_{Mg}}{V_{0}^{Mg}-Kt_{Mg}}\right)}$$
